# Temperature Field Characterization of Iron Tailings Based on Microwave Maintenance Technology

**DOI:** 10.3390/ma17020372

**Published:** 2024-01-11

**Authors:** Jun Xue, Shengjie Liu, Meng Xu, Meng Ling, Jinbao Sun, Hui Li, Xianzhang Kang

**Affiliations:** 1 Shandong Luqiao Group Co., Ltd., Jinan 250021, China; 18653165566@163.com (J.X.); 13623514137@139.com (J.S.); 13165413967@163.com (H.L.); 2 School of Civil and Transportation Engineering, Beijing University of Civil Engineering and Architecture, Beijing 100044, China; liushengjie97@126.com; 3 School of Transportation and Civil Engineering, Shandong Jiaotong University, Jinan 250357, China; 13127121042@163.com

**Keywords:** road engineering, asphalt mixture, temperature field, iron tailing, microwave heating

## Abstract

Microwave maintenance technology, as a new development trend, can realize the environmentally noninvasive and rapid repair of asphalt pavement and gradually replace traditional maintenance methods. Iron tailings were used as a self-healing material in this study to investigate the temperature response matching of microwave maintenance technology. Firstly, the physical properties and the mechanism of iron tailings were elaborated through macroscopic physical index testing and microscopic X-ray diffraction (XRD) analysis. Secondly, the applicability of aggregates to microwave heating was demonstrated by analyzing the temperature rise characteristics of the granules using infrared imaging. Then, the temperature field variation rules of the iron tailing asphalt mixture were summarized by microwave heating Marshall specimens. Finally, the road performance was assessed by conducting high-temperature dynamic stability, low-temperature tensile, water immersion Marshall, and freeze-thaw splitting tests. The experimental results showed that the iron tailings can be used as an aggregate for high-grade asphalt pavement and as the preferred aggregate for microwave maintenance technology. The iron tailings temperature field was radial from the inside out to provide different temperature response states for different pavement diseases, so the asphalt was dissolved and precipitated in a short time. The particle size of iron tailings was inversely proportional to the wave-absorbing heating rate, and the heating efficiency of the small particle size (0–4.75 mm) was the highest. The specimens doped with 4.75–13.2 mm iron tailings showed the best heating performance and road performance, with the average surface temperature of the specimens reaching 126.0 °C within 2 min. In summary, according to different disease types and construction needs, iron tailings can be used as an aggregate for asphalt pavement, providing an appropriate temperature field and improving the efficiency of the microwave maintenance of asphalt pavements.

## 1. Introduction

Cracks, potholes, and rutting are common issues that affect the structural stability and traveling comfort of asphalt pavements [1]. To address these problems, various maintenance methods have been employed. Traditional methods often involve cutting the pavement, creating construction waste, and causing traffic disruptions and increased costs [2,3]. In contrast, microwave heating maintenance technology offers a more environmentally friendly, noninvasive, and efficient repair alternative without the need for pavement cutting. Consequently, it has become a promising trend in pavement maintenance [4,5,6]. During the microwave maintenance process, asphalt does not directly absorb microwaves. Instead, the metal molecules in the aggregate of the asphalt mixture absorb the microwaves and then generate heat. This heat is then transferred to the asphalt, causing it to dissolve and resolidify, resulting in the restoration of its mechanical properties [7,8,9,10]. There are many factors affecting the mechanical properties of asphalt mixtures; temperature, aggregate properties, construction conditions, etc. have a certain influence on them. At present, many experts use nondestructive evaluation techniques for the investigate and monitor the damage, mechanical properties, cracking mechanisms, and damage development process of asphalt mixtures [11,12]. The selection of suitable wave-absorbing materials and their effective integration with microwave maintenance technology have become essential considerations in achieving optimal construction temperature fields for different pavement conditions and ensuring the longevity of the asphalt.

To enhance the microwave sensitivity of asphalt mixtures, researchers have explored the addition of high-performance wave-absorbing materials such as steel fibers, steel slag, steel chips, ferrite, and carbon nanotubes [13,14,15,16,17,18,19]. These additives facilitate the rapid warming of asphalt mixtures, as demonstrated by studies conducted by Nalbandian, Yu, Khan, and others [20,21,22]. Furthermore, modified asphalt pavements with wave-absorbing materials have shown promising healing effects and significant energy savings [23,24]. Currently, most scholars focus on optimizing the wave-absorbing performance of asphalt mixtures through the development of new materials [25,26,27]. However, they often overlook the high cost of wave-absorbing materials and the challenges associated with achieving uniform temperature distribution and aggregate gradation in the asphalt mixtures. Hence, it is necessary to identify aggregates that are cost-effective, possess good gradation, exhibit excellent wave-absorbing performance, and can be widely used in road projects to address these limitations.

Iron tailings, a byproduct of iron ore production, have attracted significant attention as a potential solution to environmental pollution and land resource occupation caused by largescale stockpiling [28,29]. Iron tailings exhibit excellent mechanical properties, including roughness, shape, angularity, hardness, polish, and abrasion resistance, making them suitable for road construction [30,31]. Previous studies by Gao, Figueiredo, and others have demonstrated the superiority of using modified iron tailings in roadbeds and their conversion into code-compliant building materials through geopolymerization technology [32,33]. Shamsi proves iron ore has excellent mechanical properties and economic benefits [34] Additionally, the recovery of valuable elements from iron tailings has been explored, increasing their reuse rate and creating economic value [35,36,37]. Despite the research conducted on the secondary resource utilization of iron tailings, their comprehensive utilization rate remains low. Most studies have focused on separating the physical and chemical properties of iron tailings [38,39,40,41], overlooking their potential as a wave-absorbing material for microwave maintenance technology. Currently, there is a lack of research on the combination of iron tailings with asphalt pavements and microwave maintenance. Therefore, the integration of iron tailings with microwave maintenance technology presents an opportunity to fully utilize their potential value while addressing environmental concerns. We systematically investigated the potential advantages of utilizing iron tailings as a self-healing material for asphalt surfaces, considering key factors such as durability, cost-effectiveness, and environmental sustainability. In contrast to conventional self-heating repair materials, iron tailings, derived as a byproduct from mining operations, present a compelling environmentally sustainable option for asphalt rehabilitation. This strategic alignment reflects the contemporary emphasis on ecologically responsible practices within the realm of construction materials, contributing to a reduction in reliance on traditional nonrenewable resources. A distinctive aspect of our study is the novel integration of the physicochemical properties of iron tailings, allowing us to explore an entirely unprecedented heat transfer mechanism. This pioneering approach involves an in-depth examination of the temperature field compatible with pavement distress conditions. Unlike previous research that primarily focused on short-term evaluations, our study pioneers a comprehensive analysis of the long-term performance and durability of asphalt pavements repaired with iron tailings. This analytical depth offers a fresh perspective on the sustained benefits derived from the application of iron tailings for asphalt rehabilitation, thereby introducing a paradigm shift in our understanding of the long-term efficacy of such materials.

In this study, we propose using the iron tailings as a new type of aggregate and investigating their compatibility with asphalt pavement and microwave maintenance technology. Through microscopic and macroscopic analysis methods, we demonstrate the wave-absorbing properties and physical characteristics of iron tailings, confirming their suitability as a high-grade asphalt concrete aggregate. Furthermore, by employing infrared thermography, we analyze the impact of temperature changes and particle properties, by preparing iron tailings specimens with different particle sizes, we observe and summarize the temperature field changes in iron tailings asphalt mixtures. Finally, by preparing Marshall specimens of iron tailings asphalt mixtures with different particle sizes, we verify and summarize their macroscopic road properties. These findings provide an experimental foundation for future research on iron tailings and microwave heating maintenance technology, as well as practical guidance for construction applications.

## 2. Materials and Methods

### 2.1. Materials

#### 2.1.1. Iron Tailings

Iron ore is mainly composed of various iron-bearing minerals. Due to the different geological conditions, iron ores are mainly categorized into limonite, magnetite, hematite, and rhodochrosite according to the chemical composition and crystal structure of the iron compounds. Different iron ores have different external forms and physical properties. Compared with other types of iron ores, such as limonite, magnetite is less reducible and usually contains some other impurity minerals. Iron tailings and basalt were selected as coarse aggregates. The selected basalt is made in Jinan, Shandong Province, China. The selected iron tailings are of the Anshan-type iron ore, which is produced in Benxi, Liaoning Province, China. The rock assemblage type is silica–iron-built, hornblende, and siliciclastic; the whole ore body is large and poor, the mineral composition of the ore is relatively simple, and it contains a high content of metallic components, with an average iron content of 27–34%. According to the standard JTG E42-2005 [42] on the coarse aggregate requirements of asphalt mixture, the physical indicators of iron tailings and basalt were tested, and the test items and results are shown in Table 1.

According to the experiment results listed in Table 1, compared with the common aggregate basalt, there is a 2.1% reduction in the crushing value of iron tailings, and the compressive capacity improves. In addition, there is an increase of 5%~10% in the apparent relative density and gross volume relative density. By contrast, the porosity decreases. In principle, the theory reflects a higher strength and a better durability performance, with all its indices meeting the requirements for road performance. Thus, iron tailings are considered applicable as high-grade asphalt pavement aggregates.

#### 2.1.2. Asphalt

The asphalt used in this experiment is SBS class Ⅰ-C modified asphalt (Shandong Luqiao Group Co., Ltd., Jinan, China), which meets the standard JTG E20-2011 [43] and the requirements of the relevant tests through the detection of the technical indicators. The test results are shown in Table 2.

#### 2.1.3. Fine Aggregate and Mineral Powder

The fine aggregates and mineral powders used to conduct the tests were made of limestone. All provided by Shandong Luqiao Group Co., Ltd. As shown in Table 3 and Table 4, the technical specifications followed by the fine aggregates and mineral powders used in these tests are all aligned with the current national standard of China, JTG E42-2005 [42].

### 2.2. Mixture Design

#### 2.2.1. Grading Design

**Figure 1:**
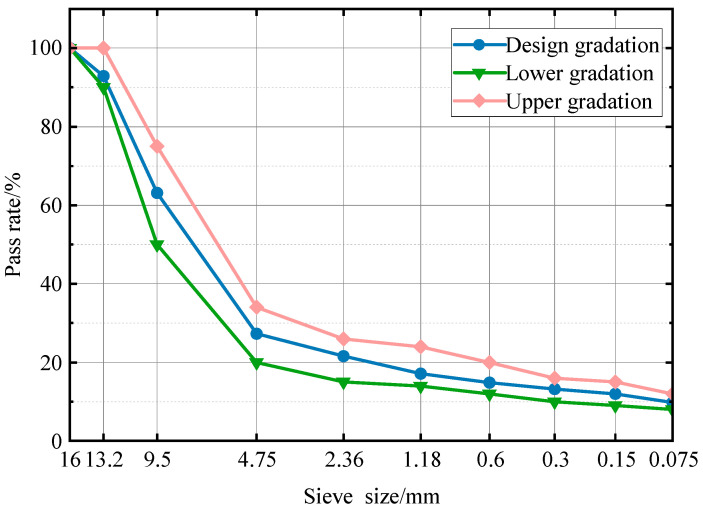
Particle size distribution of SMA-13.

The Marshall method was used to determine the proportion of basalt asphalt mixes for the control group. Figure 1 shows the selected grades. Based on past experience, a mineral aggregate gradation comparable to the mean value of the SMA-13 gradation range was selected as the target mineral aggregate.

#### 2.2.2. Optimal Asphalt Content Design

According to the requirements of the standard (JTG F40-2004) [44], the optimum asphalt mix ratio of SMA-13 basalt asphalt mixture was determined. Taking the asphalt–aggregate ratio of 6.0% as the base value, The void volume (VV), voids in mineral aggregate (VMA), voids filled with asphalt (VFA), stability, and other indicators of four groups of Marshall specimens with asphalt–aggregate ratios of 5.8%, 6.0%, 6.2%, and 6.4% were measured, as shown in Table 5. The optimum asphalt content was calculated as 6.038%. For the convenience of the test process, the optimum asphalt content of 6% was adopted in subsequent experiments.

### 2.3. Experimental Design

#### 2.3.1. Micro-Mechanical Analysis

To further explore the degree of matching between iron tailings as asphalt pavement aggregates when microwave maintenance technology was applied, the mechanism of action was demonstrated from a microscopic perspective. After the iron tailings were ground and flaked, a Leica DM2700P high-magnification microscope lens was used to analyze the internal structure through single-polarized and reflected-light observation. The iron tailings were ground using a vibrating disc grinder, and then the specimens were sieved using a sieve with 325 meshes. The physical phase analysis of the samples was performed using a D8 ADVANCE X-ray diffractometer from Bruker, Germany [45].

#### 2.3.2. Characterization of Iron Tailings

The microwave maintenance technology applied for asphalt pavement requires aggregates to perform well in wave-absorption and temperature transfer properties. For this reason, three groups of tests were conducted on the iron tailing granules.

A. The analysis was performed on the characteristics shown in terms of temperature field variation. Basalt aggregate served as the control group. Two groups of minerals (a. iron tailing, b. basalt), weighing 20 ± 0.5 g at room temperature (19 °C), were used to ensure uniform microwave heating.
B. The analysis of effect caused by granule characteristics was performed on 20 ± 0.5 g of three groups iron tailing with different particle sizes (c. 0–4.75 mm, d. 4.75–9.5 mm, e. 9.5–16 mm). Uniform microwave heating was performed for comparison tests.
C. The analysis of the effect caused by asphalt wrapping was performed. Half of the iron tailings were wrapped, using basalt as the control group, and then placed in a microwave heating environment. A thermocouple digital thermometer was used to read the temperature of the exposed part of the iron tailings and the wrapped asphalt.

In the three groups of tests above, the microwave heating instrument used was the Toshiba ER-SS20CNW. The main parameters are shown in Table 6. Sampling was performed for 3 min at an interval of 30 s. The data were collected from groups A and B using a Xinsite HTH8 infrared imager.

#### 2.3.3. Road Performance and Temperature Field Pattern

Marshall specimens were designed using the volumetric method. With basalt asphalt mixes as the reference group, the iron tailings of three different particle sizes (a. 4.75–9.5 mm; b. 4.75–13.2 mm; c. 9.5–13.2 mm) were mixed to prepare the asphalt mixture specimens according to the standard JTG E20-2011. To facilitate the continuation of the subsequent tests and in case of test errors, the total number of specimens produced in the mix design and production is shown in Table 7. Referring to the current Chinese standard JTG E20-2011, the road performance of asphalt mixture was tested, and the specific test indexes and reference protocols are as follows: the rutting (60 °C, 0.7 MPa, T0719-2011), low-temperature bending (−10 °C, 50 mm/min, T0715-2011), water immersion Marshall (T0709-2011), freeze-thaw split (T0729-2000), and three-wheel accelerated wear tests (0.7 MPa, 60 r/min). We plan to conduct the DMA test in a future study. An HTH8 infrared thermal imager was used to measure the variation in temperature rise and distribution of surface temperature within 2 min of the admixtures given different particle sizes of iron tailings and basalt Marshall specimens. The initial temperature in the four groups of Marshall specimens was restricted to the range of 19 ± 0.3 °C, and the sampling interval was set to 30 s to prevent the effect of temperature heat transfer dissipation while ensuring that the experimental environment was airtight. The Marshall specimen was separated from the microwave oven tray by placing isothermal porcelain (poor wave absorption) at the bottom of the specimen. The collected infrared thermal images of the specimen surface were processed using the HTH8 Tools software V2.0 to determine the average surface temperature of the specimen.

## 3. Results and Discussion

### 3.1. Physical Properties and Chemical Components

**Figure 2:**
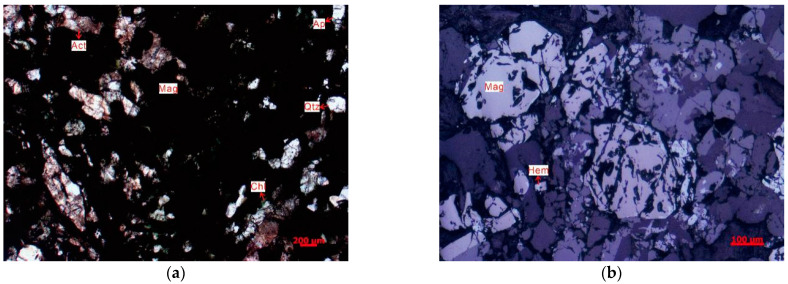
Picture of iron tailings under the microscope. (**a**) Single polarized lens photo and (**b**) reflected light microscope photo.

**Figure 3:**
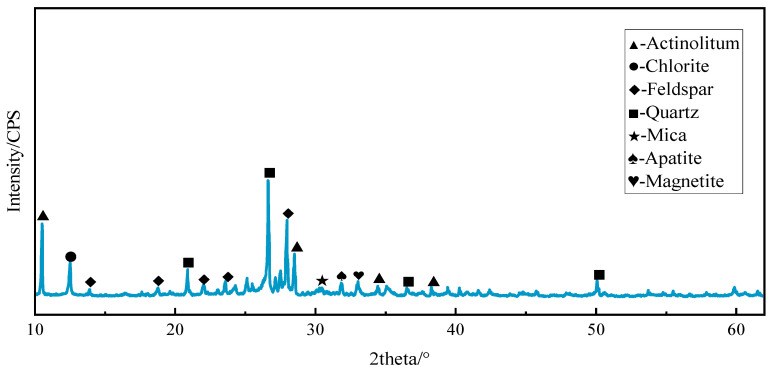
XRD results of iron tailings.

The physical properties of the iron tailings pellets were tested, as described in Section 2.1.1. Figure 2 shows the findings of microscopic observation, and Figure 3 shows the image of XRD diffraction for iron tailings.

The mineralogical composition of the iron tailings employed in our investigation, as depicted in Figure 2 and Figure 3, reveals a diverse range of minerals, including magnetite (Mag), actinolite (Act), quartz (Qtz), chlorite (Chl), apatite (Ap), hematite (Hem), mica, feldspar, and other constituents. Notably, magnetite emerges as the predominant metallic mineral, exhibiting commendable crystallization properties and constituting a substantial proportion of the overall mineral content in the iron tailings. The prevalence of magnetite in the mineral assemblage suggests pronounced magnetic characteristics within the iron tailings. This observation leads us to infer that the material possesses inherent magnetic properties, an attribute that holds significance in the context of microwave maintenance technology. Specifically, owing to the abundance of magnetite, which has well-defined crystalline structures, the iron tailings are expected to exhibit notable wave absorption capabilities within a microwave environment.

Consequently, our findings support the contention that iron tailings can serve as a preferred aggregate for microwave maintenance technology. The magnetic properties conferred by the abundance of magnetite imply the potential for effective wave absorption, positioning iron tailings as a viable and advantageous choice for applications in microwave-assisted pavement maintenance. This aligns with the premise that the composition and characteristics of iron tailings make them conducive to facilitating microwave-induced repairs in asphalt surfaces.

### 3.2. Analysis of Microwave Properties of Iron Tailing Pellets

#### 3.2.1. Analysis of Temperature Change Characteristics

**Figure 4:**
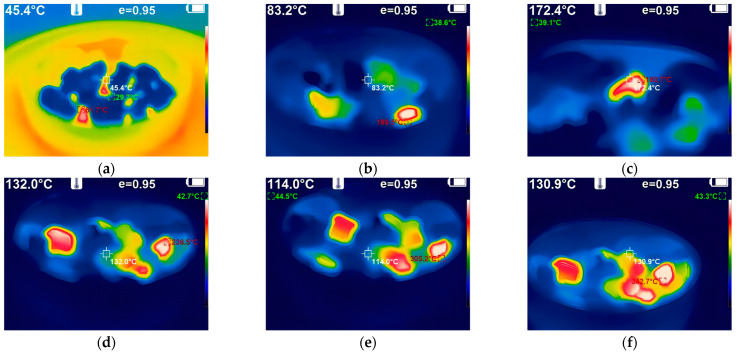
Infrared thermal imager sampling map of aggregates. (**a**) Heating 30 s; (**b**) Heating 60 s; (**c**) Heating 90 s; (**d**) Heating 120 s; (**e**) Heating 150 s; (**f**) Heating 180 s.

Figure 4a–f show the heating state of iron tailings with basalt in a microwave environment in the group A tests in Section 2.3.2. The confidence level (95%) for the presented data is represented as “e = 0.95”.

The examination of Figure 4a–f elucidates the material absorption heating states under microwave exposure. When subjected to a 30 s microwave treatment at an identical initial temperature, the iron tailings demonstrated a significant temperature elevation exceeding 61 °C, contrasting with the marginal increase of approximately 10 °C observed in basalt aggregates. Subsequently, following a 60 s exposure, the central temperature of the iron tailings surpassed 170 °C, marginally exceeding the temperature at the aggregate’s periphery by 155 °C. Remarkably, the temperature gradient remained relatively constant, and the radial temperature evolution within the iron tailings exhibited a consistent increase from the interior to the exterior. This behavior can be attributed to the rapid movement of dielectric molecules within the iron tailings induced by the microwave electromagnetic field, facilitating a microscopic and uniform heating pattern throughout the spatial entity.

Further observations revealed that the temperature of the iron tailings exceeded 300 °C, a notable contrast to the modest rise to approximately 80 °C observed in ordinary basalt. This discrepancy underscores the remarkable efficiency of iron tailings in energy utilization during microwave heating, with the temperature of the iron tailings reaching 3–4 times that of basalt.

A comprehensive analysis of these results supports the assertion that iron tailings exhibit elevated energy utilization efficiency, swift absorption of microwave heating, and uniform temperature distribution throughout the microwave exposure process. This collective behavior significantly abbreviates the required heating duration, thereby enhancing the overall construction efficiency of microwave-assisted pavement maintenance. The observed temperature profiles underscore the potential of iron tailings as an efficient and effective aggregate for applications in microwave-based repair technologies.

#### 3.2.2. Analysis of the Influence of Granule Characteristics

**Figure 5:**
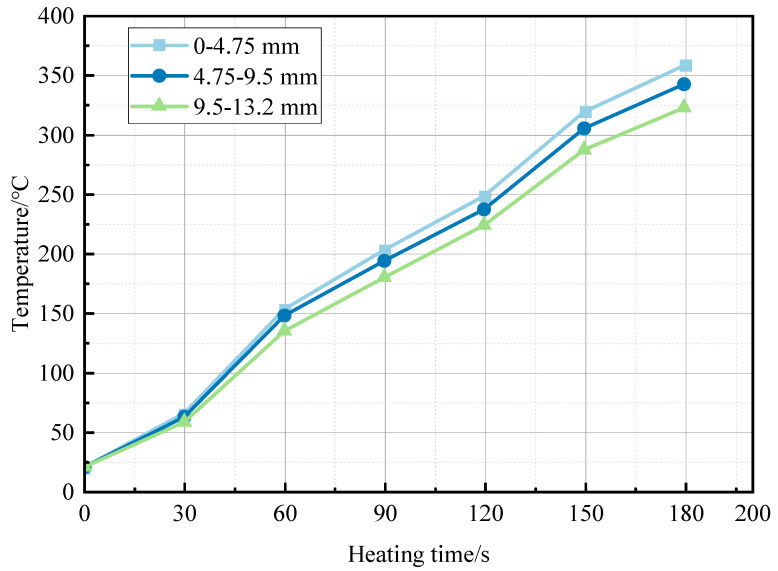
Iron tailings with different particle size heating.

Figure 5 shows the test data of group B, as described in Section 2.3.2.

According to experimental results presented in Figure 5, the particles with different sizes behave in the same way in terms of heating by microwave absorption, The reason is that the internal structure of iron tailings is proportional to the particle size, which makes the impact of particle size on absorption and warming performance less apparent. However, as particle size increases, the heating rate gradually decreases. The heating efficiency is highest for small particles (0–4.75 mm), reaching 113 °C/min, which is about 5% higher than that of medium-sized particles and 12% higher than that of coarse particles. The dielectric constant of the material is constant, and the temperature difference is due to the increase in particle size, which reduces the effective area of aggregate subjected to microwave radiation, resulting in the decrease in its microwave absorption capacity and the decrease in the heating rate. In actual construction, we should comprehensively consider the particle size distribution and the applicability of microwave conservation, and choose the best proportion.

#### 3.2.3. Analysis of the Impact of Bitumen-Coated Iron Tailings

**Figure 6:**
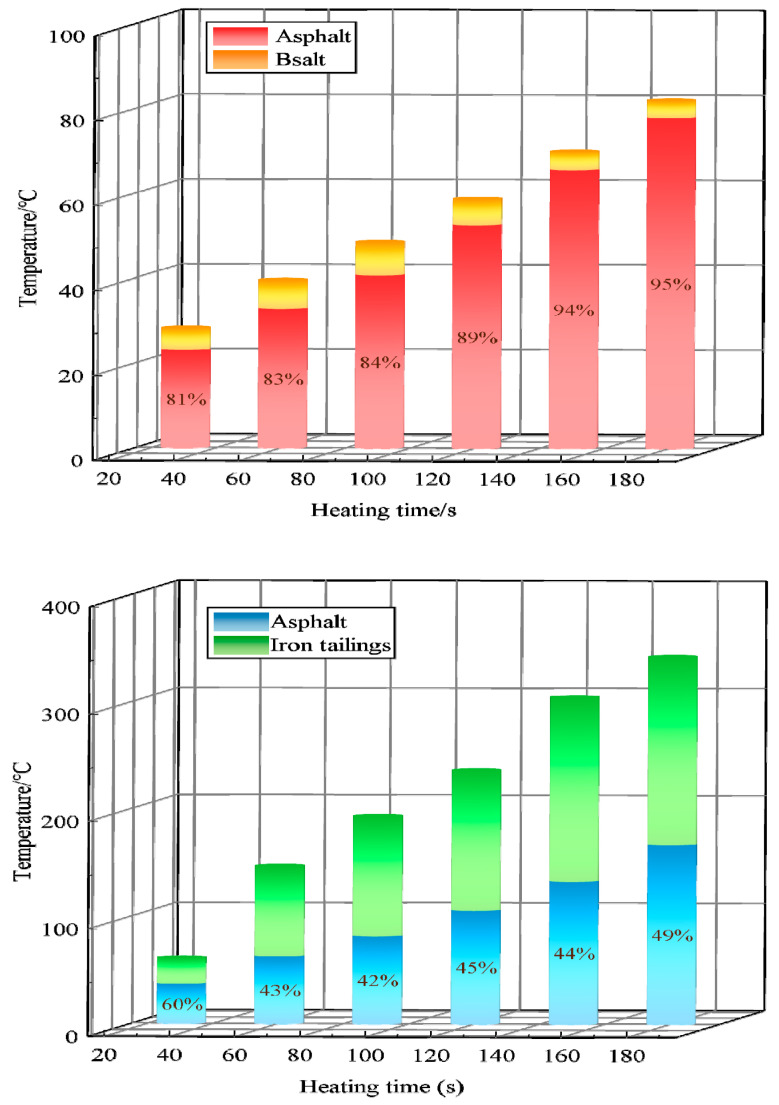
Temperature rise diagram of asphalt-coated aggregates.

There is also a significant difference in the capacity of wave absorption between asphalt and aggregate in the presence of microwaves. To explore the performance of the aggregate in heat transfer to asphalt during construction, the group C tests described in Section 2.3.2 were analyzed. Figure 6 demonstrates the impact of heating basalt and iron tailings on the heat transfer to asphalt.

According to the comprehensive analysis shown in Figure 6, there is no significant difference in temperature between basalt aggregates and asphalt. However, the overall pace of temperature rise is slow, and the temperature difference shows consistency. Upon analysis, it can be determined that this is due to the fact that asphalt and basalt both contain very few polar metal molecules, thus leading to a slow warming pace. The final asphalt temperature reached a low of 77.9 °C. The comparison between iron tailings and basalt shows the characteristics of extremely strong wave-absorption warming, with a significant difference between asphalt and aggregate temperatures. However, asphalt temperature also shows regular growth. At 30 s, when the asphalt was in an optimal heating load state, its temperature reached about 60% of the surface temperature of iron tailings. This is due to the initial heating conditions, in which a small number of asphalt’s polar molecules interact with the basalt surface, enhancing the magnetite and wave absorption heating activity. This results in relatively fast asphalt warming. Subsequently, the asphalt temperature reached about 45% of the temperature of iron tailings. Considering the analysis described in Section 3.2.1, it is known that this is because the temperature field of iron tailing is radiating from the inside out. Therefore, the temperature difference between asphalt and basalt gradually stabilizes. However, the asphalt temperature increased far more rapidly than the wrapped basalt. It reached 167.4 °C when heated for 3 min, which is about two times that of the wrapped basalt.

Iron tailing shows excellent heat transfer performance to asphalt, which meets the requirements of microwave heating maintenance technology for aggregate. In spite of this, this is only a preliminary judgment made on a single aggregate. In the next phase, it is necessary to explore the actual pavement structure by considering the state of the microwave heating law of authenticity.

### 3.3. Asphalt Mixture Temperature Field Characteristics Study

**Figure 7:**
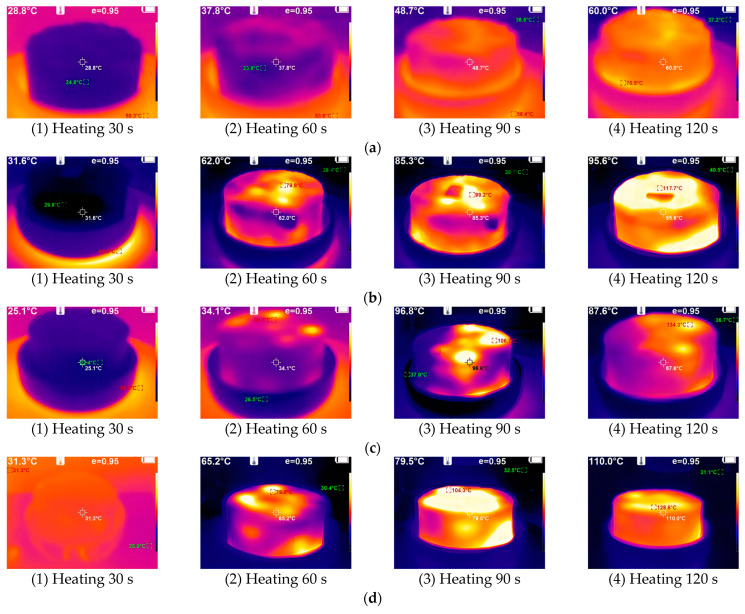
Infrared imaging of Marshall specimen. (**a**) Ordinary basalt Marshall specimen; (**b**) Marshall specimen of iron tailings (4.75–9.5 mm); (**c**) Marshall specimen of iron tailings (9.5–13.2 mm); (**d**) Marshall specimen of iron tailings (4.75–13.2 mm).

**Figure 8:**
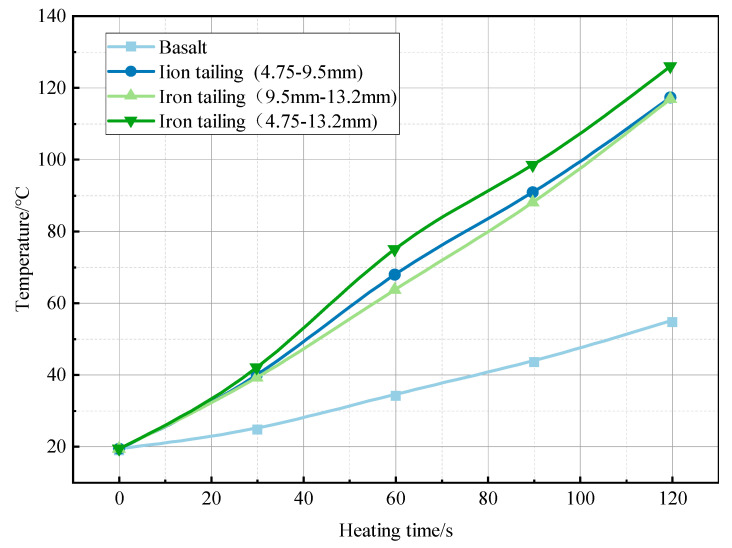
Asphalt mixture surface temperature.

**Figure 9:**
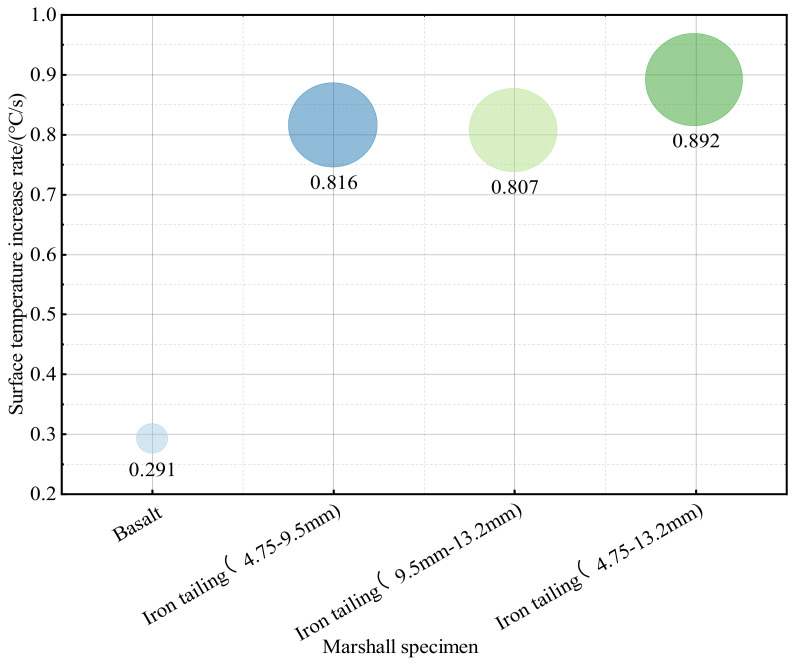
Heating rate graph.

Further exploration of the response state of asphalt pavement to microwave heating technology was necessary to provide the appropriate temperature for different construction conditions and pavement diseases. The specific experimental results are given in Figure 7, Figure 8 and Figure 9.

As shown in Figure 7, Figure 8 and Figure 9, the Marshall specimens mixed with different grades of iron tailings showed significant warming characteristics. The basalt specimen surface heating trend was relatively slow because basalt contains fewer metal mineral components and polar molecules, leading to its weak microwave absorption ability. Consequently, the heating phenomenon was less pronounced. Additionally, the lower efficiency of heat transfer results in a surface heating rate that is much lower than that of the specimen doped with iron tailings. The specimens mixed with 4.75–9.5 mm iron tailings reached 117 °C after heating for 2 min, and the surface temperature was relatively uniform. The temperature of the specimens mixed with 9.5–13.2 mm iron tailings reached the maximum value of 134 °C, but the surface temperature distribution was not uniform due to the slow increase in the temperature of fine aggregates, with an average surface temperature of 116.8 °C. The specimens mixed with 4.75–13.2 mm iron tailings had a stable warming trend and the most uniform surface temperature. After 2 min, the average temperature reached 126.0 °C, and the average surface temperature increase rate reached a maximum value of 0.892 °C/s. This proved that the higher the content of wave-absorbing aggregates, the faster the warming rate. From the temperature rise curve, the temperature rise of the blended specimens with fine gradation and 4.5–13.2 mm blended specimens was more stable.

Combined with the analysis of the temperature rise characteristics of iron tailings pellets in Section 3.2, it has been demonstrated that the finer the blended aggregate, the more stable the temperature rise tends to be and the more uniform the surface temperature distribution is. Although the heating effect of the basalt specimen was not as good as that of doped specimen, it also shows good heating characteristics in the microwave environment in combination with the usual traditional heating test, which proves that the microwave heating technology has great advantages in the road thermal regeneration and the eradication of pavement diseases.

### 3.4. Road Performance of Iron Ore Tailing Asphalt Mixes

**Figure 10:**
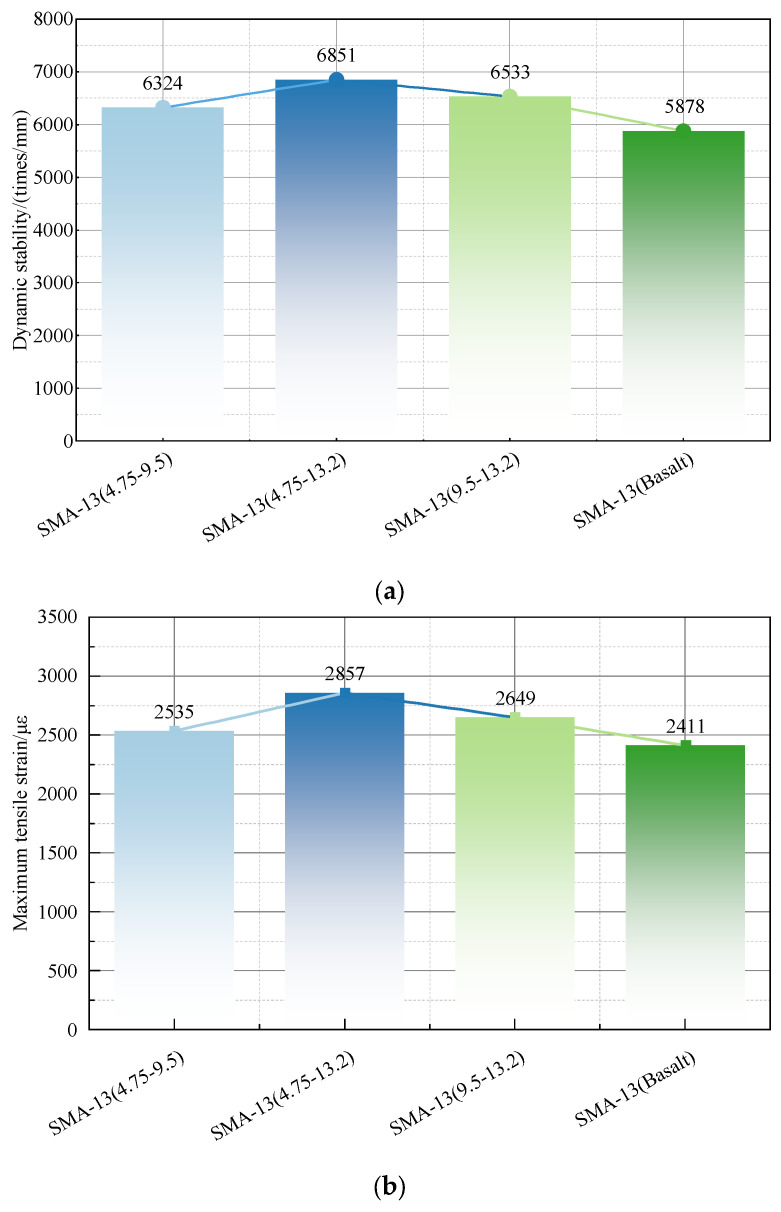

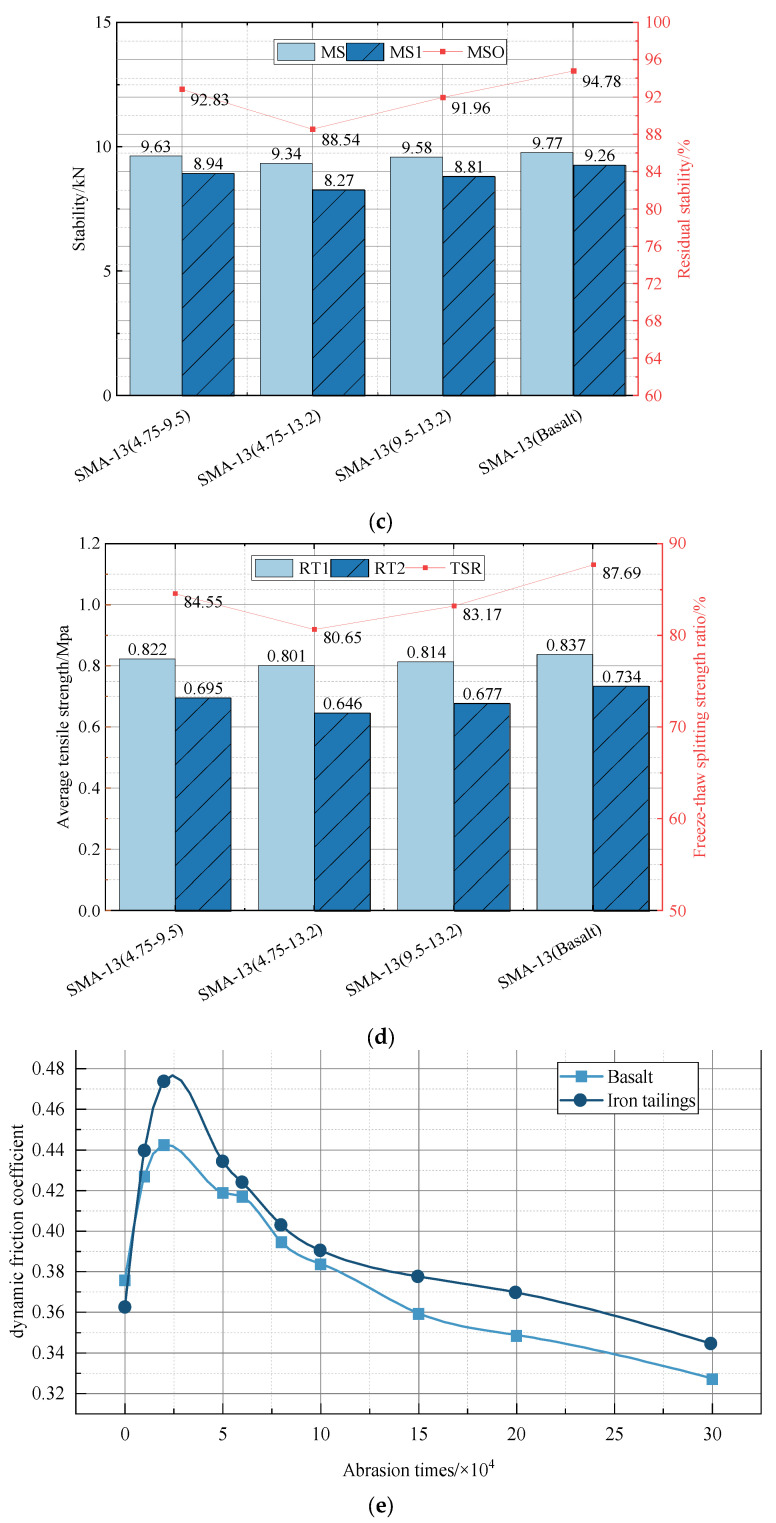
Iron tailings road performance. (**a**) Dynamic stability results of different mixtures. (**b**) Maximum bending tensile strain. (**c**) Water immersion Marshall test. (**d**) Freeze-thaw splitting test. (**e**) Three-round accelerated loading test.

To investigate the road performance of the iron tailing asphalt mixture, the test specimens were prepared by mixing and replacing the iron tailings of different particle sizes, as described in Section 2.3.3. The high temperature stability was evaluated using the dynamic stability index in a high-temperature rutting test. The low-temperature performance was evaluated using low-temperature bending maximum tensile strain in −15 °C, the water stability was evaluated using residual stability and the TSR index in an immersion Marshall test and the freeze-thaw splitting test, and the wear resistance performance was evaluated using the dynamic friction tester (DFT) in a three-round accelerated loading test. The test results are shown in Figure 10a–e.

Figure 10a–e show that:

(1) The asphalt mixture supplemented with the iron tail ore with particle sizes of 4.75–13.2 mm performs best in high-temperature stability, and the dynamic stability reaches 6851 times/min, which is 16.55% higher than basalt. This is because iron tail ore outperforms ordinary basalt in angularity and roughness, which meets the requirements of the road performance. Therefore, it is applicable in the construction of pavement that the iron tailing granular material with a wide range of gradation and good grade should be selected for actual construction.
(2) The asphalt mixture supplemented with iron tail ore demonstrates greater low-temperature stability. The critical strain caused by damage is 1.05~1.18 times that of basalt, and low-temperature stability is improved compared with lighter basalt binder asphalt components. As a result, the flexibility of the asphalt mixture is enhanced. Therefore, the iron tail ore asphalt mixture meets the road performance requirements on low-temperature stability.
(3) According to the test results, the incorporation of iron tail ore affects the water stability of asphalt mixes to some extent, and the residual stability of iron tail ore asphalt mixes with a particle size of 4.75–13.2 mm is reduced to 88.54%, which is 6.58% lower than basalt. In addition, the ratio of splitting strength to freeze-thaw splitting strength is reduced by 8%, which is because the quartz and mica content in iron tail ore is more comparable to basalt, and the degree of crystallization is higher. Despite a reduction in the water stability of the iron tail ore asphalt mixture, it still meets the standard JTG F40-2004, has a residual stability over 80% in the Marshall test, and even meets the requirements of the wet area of not less than 85%. Therefore, the iron tail ore asphalt mixture meets the requirements of construction on water stability and can be applied to road construction.
(4) The peak friction coefficient of iron tailing is 0.47, which is a slight increase compared with the 0.44 of basalt. It peaked and then declined, showing a trend of gradual stabilization until 150,000 iterations. In addition, the difference between these two friction coefficients stabilized at about 0.02. In contrast, the iron tailing ore asphalt mixture continued demonstrating better anti-slip properties than those of the basalt asphalt mixture, which meets the relevant requirements.

According to a comprehensive analysis of the road performance of iron tailings asphalt mixtures, iron tailing blending reflects the variability of road performance. However, it should be noted that the results meet the current construction specifications for higher asphalt pavement requirements (JTG F40-2004). It is demonstrated that iron tailing can replace basalt as an asphalt aggregate for use on high-grade road surfaces.

## 4. Conclusions

In this paper, a low-cost and simple-process iron tailing asphalt aggregate is proposed, which can provide the corresponding temperature field for different pavement diseases and construction needs. By studying the properties of iron tailing pellet components, the law of wave absorption, and temperature rise, asphalt mixture design and temperature field distribution have been determined. The main conclusions obtained are as follows:

1. Iron tailing aggregate can be used for the paving asphalt mixture of high-grade pavement. Iron tailings contain a large amount of metallic mineral components (i.e., magnetite) and have a certain degree of magnetic properties and wave absorbing ability in microwave maintenance technology.
2. The temperature field of iron tailings radiates from inside out, and there is a balance reached in heat transfer. The asphalt can be dissolved before rapid precipitation. The particle size of iron tailings was inversely proportional to the wave-absorbing heating rate. Moreover, heating efficiency is the highest for small-sized particles (0–4.75 mm), reaching 113 °C/min.
3. The iron tailing asphalt mixture supplemented with iron tailings (4.5–13.2 mm) meets long-term road performance requirements. Compared with basalt, the high-temperature dynamic stability is improved by 16.55%, the maximum low-temperature tensile strain reached 1.18 times the original level, and there was a reduction in water stability, despite meeting the relevant requirements.
4. The specimen mixed with iron tailings with particle sizes of 4.75–13.2 mm displayed a consistent warming trend and uniform distribution of surface temperature, which can provide different temperature response states for different pavement diseases.

This study is currently in the laboratory test stage. Because the actual generation mechanism of road disease and maintenance environment are complicated, the onsite test is of great necessity. Therefore, in the next step, we will lay a certain length of experimental sections in different environments to investigate and verify the effects of microwave maintenance on iron tailing asphalt mixtures and analyze the degree of asphalt aging.

## Figures and Tables

**Table 1:** Properties of coarse aggregate.

Index	Technical Quality Requirements	5~10	10~15	Basalt	Test Method
Aggregate crushing value/%	≤26	-	18.2	20.3	JTG E42 T0316
Apparent relative density	≥2.6	2.813	2.886	2.621	JTG E42 T0304
Gross volume relative density	Actual measurement records	2.726	2.834	2.596	JTG E42 T0304
Water absorption rate/%	≤2.0	1.32	0.49	1.1	JTG E42 T0304
<0.075 mm particle content/%	≤1.0	0.08	0.01	0.56	JTG E42 T0310

**Table 2:** Properties of SBS-modified asphalt.

Index		Unit	Technical Quality Requirements	Result	Test Method
Needle penetration (25 °C)		mm	60–80	70.7	JTG E20 T0604
Needle penetration index PI		-	−0.4~1.0	0.46	JTG E20 T0604
Softening point (R and B)		°C	≥55	68	JTG E20 T0606
Latency (5 cm/min, 5 °C)		cm	≥30	51	JTG E20 T0605
Kinematic viscosity (235 °C)		Pa.s	≤3	1.9	JTG E20 T0625
Resilient recovery (25 °C)		%	≥65	79	JTG E20 T0662
Softening point difference		°C	≤2.5	1.6	JTG E20 T0661
Residues after RTFOT	Quality change	%	≤±1.0	−0.07	JTG E20 T0610
	Needle penetration ratio	%	≥60	81	JTG E20 T0604
	Residual latency (15 °C)	cm	≥20	34	JTG E20 T0605

**Table 3:** Properties of fine aggregate.

Index	Unit	Technical Quality Requirements	Test Result	Test Method
Apparent density	g/cm^3^	≥2.50	2.813	JTG E42 T0328
Angularity	s	≥30	49.2	JTG E42 T0345
Sand equivalent	%	≤3	1.37	JTG E42 T0334

**Table 4:** Properties of mineral powder.

Index		Unit	Technical Quality Requirements	Test Result	Test Method
Apparent density		g/cm^3^	≥2.50	2.717	JTG E42 T0352
Water content		%	≤1	0.19	JTG E42 T0103
Particle size range	<0.6 mm	%	100	100	JTG E42 T0351
	<0.15 mm		90~100	94.3	
	<0.075 mm		75~100	80.3	
Hydrophilic coefficient		-	<1	0.69	JTG E42 T0353
Plasticity index		-	<4	2.4	JTG E42 T0355

**Table 5:** Basalt asphalt mixture test.

Grading Type	Asphalt Dosage/%	Void Ratio VV/%	Gap Rate VMA/%	Saturation VFA/%	Stability/kN	Gross Volume Relative Density/(g/cm^3^)	Theoretical Maximum Relative Density/(g/cm^3^)
SMA-13	5.8	4.0	18.3	78.7	10.87	2.637	2.757
	6.0	3.4	17.6	80.1	11.40	2.655	2.740
	6.2	3.2	17.4	83.6	13.28	2.674	2.728
	6.4	2.8	16.7	86.3	11.51	2.668	2.714
Technical requirements	-	3~4.5	≥16.5	70~85	≥6.0	-	-

**Table 6:** Microwave heating apparatus main parameters.

Volume	Rated Voltage/Frequency	Microwave Input Power	Microwave Output Power	Microwave Frequency	Overall Dimensions (mm)	Oven Cavity Size (mm)	Product Net Weight
20L	220 V/50 Hz	1270 W	800 W	2450 MHz	440 × 330 × 259	306 × 304 × 206	1.2 kg

**Table 7:** Number of specimens.

Specimen Type	Basalt	Iron Tailings	Iron Tailings	Iron Tailings
Particle size (mm)	-	4.75–9.5	4.75–13.2	9.5–13.2
Quantity	2	4	4	6

## Data Availability

Some or all data or codes generated or used during this study are available from the corresponding author by request.
